# Active Aging for L.I.F.E.: An Intergenerational Program to Improve Adolescents’ Aging Attitudes in Rural Communities

**DOI:** 10.3390/ijerph23070822

**Published:** 2026-06-23

**Authors:** Xuewei Chen, Emily Roberts

**Affiliations:** 1 Department of Biomedical & Digital Health Intelligence in Clinical Systems, Institute of Medical Artificial Intelligence, College of Osteopathic Medicine, Sam Houston State University, Conroe, TX 77304, USA; 2 College of Education and Human Sciences, Oklahoma State University, Stillwater, OK 74078, USA; emily.roberts12@okstate.edu

**Keywords:** rural health, intergenerational programs, adolescence, aging attitudes, health literacy, preventive health, school-based intervention, community capacity building

## Abstract

**Highlights:**

**Public health relevance—How does this work relate to a public health issue?**

- Rural adolescents experience limited access to preventive health education and intergenerational health resources, contributing to long-term rural health inequities.
- This study evaluates a school-based, intergenerational program delivered within rural health and education settings, addressing social determinants of health across the life course.

**Public health significance—Why is this work of significance to public health?**

- An intergenerational, train-the-trainer model involving older adults and college students significantly improved rural adolescents’ aging attitudes, health awareness, and social connectedness.
- Findings demonstrate that brief, low-cost interventions embedded in existing rural school curricula can reduce sex/gender- and age-based gaps in health-related perceptions.

**Public health implications—What are the key implications or messages for practitioners, policy makers and/or researchers in public health?**

- Rural health services and school systems can leverage intergenerational partnerships to expand preventive health education and workforce capacity without reliance on clinical infrastructure.
- Scalable programs like Active Aging for L.I.F.E. support rural health service innovation by strengthening early prevention, community engagement, and lifespan health literacy.

**Abstract:**

Rural adolescents face persistent health inequities driven by limited access to preventive health education, intergenerational engagement, and resources that support lifelong wellness. This study evaluated the effectiveness of Active Aging for L.I.F.E., a school-based intergenerational health literacy program, in improving adolescents’ attitudes toward aging and health. The four-session program, delivered through a train-the-trainer model involving older adults and undergraduate students, was implemented in three rural schools during the 2024–2025 academic year. A total of 86 junior high and high school students participated, with 77 completing pre- and post-program surveys assessing attitudes toward aging, health consciousness, and intergenerational engagement. Paired *t*-tests and multiple regression analyses examined overall program effects and differences by sex/gender and age group. Students demonstrated significant improvements in aging attitudes, perceived relevance of aging topics, enjoyment of intergenerational interaction, and awareness of health-promoting behaviors across the lifespan. Several baseline sex/gender and age-based gaps in health-related perceptions were reduced following participation, with stronger future-oriented attitude shifts observed among younger adolescents. These findings suggest that brief, scalable intergenerational interventions embedded in rural school settings can support early prevention, health literacy, and community capacity building, offering a promising strategy for advancing rural public health outcomes across the life course.

## 1. Introduction

People living in rural communities face heightened risks for adverse physical and mental health outcomes due to persistent barriers in healthcare access, socioeconomic instability, and geographic isolation. According to the Centers for Disease Control and Prevention, rural populations experience higher rates of chronic disease, poorer mental health outcomes, and reduced access to preventive health services compared to their urban counterparts. In Oklahoma, where more than one-third of residents live in rural areas, these disparities underscore the importance of school-based interventions that can reach youth early and address broader social and environmental determinants of health [1].

Intergenerational health literacy interventions may offer a promising strategy for addressing these challenges by simultaneously promoting adolescent health awareness and strengthening community social capital. The Active Aging for L.I.F.E. program was supported through a grant from the NEXT50 Initiative, a Denver-based organization that funds projects that address new ways of thinking about the aging process, and was developed through outcomes from a previous pilot study [2,3].

Active Aging was termed by the World Health Organization in the early 1990s to provide evidence that better physical function, mental health, and social health can be achieved from shifting the research focus on chronic disease outcomes to more relevant outcomes that affect independence and quality of life [4]. While Active Aging programs in the past have solely addressed the needs of older adults 65+, this program brings together three generations, older adults, college-age students, and adolescent school-age students [5].

Empirical evidence from prior evaluations of the Active Aging for L.I.F.E. program further underscores the value of intergenerational engagement for influencing attitudes and behaviors related to aging across the life course. Interactions between younger and older participants facilitated bidirectional learning, where younger individuals reported increased awareness of how present health behaviors shape future aging outcomes, while older adults shared lived experiences and, in turn, reflected on strategies to enhance their own well-being [2,3]. In a pilot pre–post study with 20 college students and 23 older adults, college-aged participants demonstrated significant attitudinal shifts, including decreased endorsement of the belief that “aging is a time of loss” and increased recognition of the roles of environment, independence, and cognitive activity in healthy aging, with both groups reporting that program content was personally relevant [5]. Follow-up focus groups conducted six months after program completion further indicated sustained impacts, as participants from both generations described changes in attitudes and behaviors, such as adopting proactive coping strategies, prioritizing social connectedness, and sharing knowledge with family members; younger participants reported taking steps to avoid social isolation and thinking about aging in new ways, while older adults emphasized greater self-awareness and adaptive health behaviors [6]. Collectively, these findings highlight how intergenerational programming not only enhances knowledge and perceptions of aging but also promotes reciprocal learning, behavior change, and the diffusion of health information across generations.

All program participants were introduced to factors relating to social connectedness, mindfulness, and disease prevention as they relate to wellness across the lifespan. Central to the intervention is the intergenerational structure of the L.I.F.E. leader groups, who share the program with the adolescents, allowing for conversations between generations. These interactions support relationship building, challenge age-based stereotypes across generations, and foster mutual understanding of resilience and health across the life course. Such opportunities are particularly valuable for adolescents growing up in rural communities, where access to mental health resources, preventive health education, and positive intergenerational engagement may be limited.

Both the L.I.F.E. leader training group and the school outreach students participated in a variety of presentations on the L.I.F.E. framework, which includes the four modules of Longevity, Independence, Fitness, and Engagement. In addition, there were group activities, which included small group discussions about how to integrate Longevity factors in the International Blue Zone into daily patterns of life, a empathy exercise with young adults/adolescents using wheelchairs and walkers while wearing Gerontological GERT simulation equipment, mindfulness small group discussions about how to navigate difficult mental health situations to build resilience, and an individual short written reflection entitled “See Me at 70” where students were asked to describe the beliefs, values, and life accomplishments of their 70-year-old future self.

The purpose of this study was to evaluate the effectiveness of this Active Aging for L.I.F.E. program among junior high and high school students and to determine whether program-related changes varied by students’ sex/gender or age group. Accordingly, we addressed the following research questions:

(1) Did students’ beliefs and attitudes toward aging and health change significantly after participating in the four-part program?

(2) Did these changes differ by sex/gender?

(3) Did these changes differ by age group?

## 2. Materials and Methods

### 2.1. Procedures and Participants

The Active Aging for L.I.F.E. program was implemented in three rural schools during the 2024–2025 academic year. Prior to program delivery, a team of 22 older adults (aged 51 to 85) and 26 undergraduate students (aged 18 to 35) participated in the train-the-trainer workshops covering the program’s four core sessions: Longevity, Independence, Fitness, and Engagement. All older adults were Caucasian, and the majority of the college students were also Caucasian. Snowball sampling was used to recruit L.I.F.E. Leaders. Inclusion criteria for the college students included being currently enrolled in college, aged 18 or older, and able to sit and/or walk for extended periods of time. For older adults, eligibility criteria included being aged 50 or older and able to sit and/or walk for extended periods of time.

Each training session lasted approximately 90 min. After completing the workshops, the trained intergenerational L.I.F.E. leaders delivered the same four-session curriculum to adolescents in classroom settings during regular school hours. Each of the four in-class sessions took place in a 50 min class period. The four-part program times and dates were pre-planned with the classroom teachers. All of the classes were within the Family and Consumer Science curriculum at the schools.

A total of 86 students participated in the program, including 45 junior high school and 41 high school students. Participation was voluntary, and parental consent and student assent were obtained in accordance with institutional review board approval. Paper-and-pencil surveys were administered by trained research personnel, with assistance from classroom teachers, to ensure confidentiality and consistency in data collection procedures.

### 2.2. Measures

#### 2.2.1. Aging Attitudes and Beliefs

Students’ attitudes toward aging were assessed using a set of statements rated on a 5-point Likert scale ranging from 1 = strongly disagree to 5 = strongly agree. Items captured multiple domains relevant to the Active Aging for L.I.F.E. curriculum, including attitudes toward aging as gain, relevance of aging-related topics, enjoyment of intergenerational engagement, personal health consciousness, and perceptions of aging as loss. Example statements included “It is a privilege to grow old”, “Topics related to active aging are relevant to me”, “I like spending time with older adults aged 65 and older”, “Brain exercise is as important as physical exercise for my well-being”, and “Aging restricts involvement in society”. All items were adapted from previously developed measures used in earlier iterations of the Active Aging for L.I.F.E. program, which demonstrated acceptable reliability. Details regarding the measure development process and associated factor analyses are available in our previous publication [7].

#### 2.2.2. Demographics

Participants self-reported their sex/gender (male or female). Because the survey combined sex and gender into a single item, we refer to this variable as sex/gender. Other demographic variables included age, race/ethnicity (White, African American, American Indian, Hispanic, Asian, and Other), and grade level (7th grade, 8th grade, freshman high school, sophomore high school, junior high school, and senior high school).

### 2.3. Data Analysis

Paper-and-pencil survey data was entered into Excel and subsequently imported to Stata/MP 19 for data analysis. Descriptive statistics were first examined to evaluate data quality and sample characteristics. To assess within-person changes in attitudes toward aging from pre- to post-program, paired *t*-tests were conducted. The paired *t*-test was selected because the study design involved repeated measurements from the same participants, resulting in dependent observations. This approach appropriately accounts for within-subject correlation and tests whether the mean change over time differs significantly from zero [8]. To examine whether changes differed by demographic factors, multiple regression models were estimated with change scores as the dependent variables; sex/gender and age group were treated as predictor variables. Statistical significance was defined as *p* < 0.05 for all analyses.

## 3. Results

### 3.1. Participants’ Demographic Characteristics

As shown in Table 1, a total of 86 adolescents participated in the study. Students ranged in age from 11 to 18 years old, with a mean age of 14.26 years (SD = 1.85). The sample included 53 girls (62%) and 33 boys (38%). Participants represented a diverse range of racial and ethnic backgrounds. The largest group identified as White (44%), followed by Hispanic (19%), American Indian (11%), and smaller proportions identifying as African American (5%), Asian (2%), mixed-race identities (16% combined across listed categories), or other race (2%). One student did not report race/ethnicity. Students were distributed across grade levels as follows: 7th grade (26%), 8th grade (27%), 9th grade/freshman (22%), 10th grade/sophomore (10%), 11th grade/junior (10%), and 12th grade/senior (5%).

### 3.2. Did Students’ Beliefs and Attitudes Toward Aging and Health Change Significantly After Participating in the Program?

There were 77 students who completed both the pre-program and post-program surveys assessing attitudes and beliefs about aging. Students demonstrated significant positive changes in beliefs and attitudes toward aging and health following participation in the Active Aging for L.I.F.E. program. As shown in Table 2, they reported stronger agreement with a key indicator of attitudes toward aging as gain, endorsing more strongly the statement “It is a privilege to grow old” (t(73) = 3.24, *p* = 0.002). Students also reported greater perceived relevance of program topics, as evidenced by increased agreement with “Topics related to active aging are relevant to me” (t(71) = 2.49, *p* = 0.015), along with heightened enjoyment of intergenerational engagement, reflected in increased agreement with “I like spending time with older adults aged 65 and older” (t(74) = 2.16, *p* = 0.034).

Students also exhibited significant gains in personal health-consciousness, including stronger endorsement of the importance of face-to-face social connections (t(73) = 2.58, *p* = 0.012), the value of engaging in brain exercise (t(72) = 3.00, *p* = 0.004), and the role of their living environment in supporting independence (t(73) = 2.11, *p* = 0.039). Additionally, students reported reduced difficulty discussing emotions, indicated by a significant decrease in agreement with the statement “Discussing my feelings is difficult for me” (t(73) = −2.21, *p* = 0.030).

With respect to attitudes toward aging as loss, students became more likely to acknowledge age-related challenges after the program, including reduced societal involvement (t(73) = 2.58, *p* = 0.012) and spending less time with friends and family in later life (t(74) = 2.79, *p* = 0.007). Taken together, these findings indicate that the program effectively enhanced students’ aging awareness, health consciousness, and comfort with emotional communication.

### 3.3. Did These Changes Differ by Sex/Gender?

Analyses indicated that program-related changes differed by sex/gender for at least one key outcome (Table 3). Female students showed a significantly greater increase than male students in recognizing the importance of exercising at any age (*p* = 0.047). At baseline, female students were less likely than male students to endorse this statement (*p* = 0.028); however, this difference was no longer significant after the program, suggesting that the intervention helped close the initial gender gap in this health-related belief.

Several additional baseline sex/gender differences were observed. Prior to the intervention, female students were also less likely to agree that they enjoyed spending time with older adults age 65 and older (*p* = 0.008) and less likely to agree that “My life has purpose” (*p* = 0.032); however, male students were less likely than females students to agree that “Topics related to active aging are relevant to me” (*p* = 0.009). Following the intervention, these baseline sex/gender differences were no longer observed. At post-intervention, female students were more likely than males to agree that “My personal behaviors such as sleep and eating patterns impact my health” (*p* = 0.036) compared to male students. Across other domains, changes were generally in the expected direction for both groups, and no additional statistically significant sex/gender differences were observed.

### 3.4. Did These Changes Differ by Age Group?

Significant differences by age group were observed. Junior high school students demonstrated a greater increase than high school students in looking forward to growing older (*p* = 0.009), indicating a stronger improvement in future-oriented attitudes about aging among younger adolescents. At baseline, compared to high school students, junior high school students were more likely to agree that “Quality of life declines as people age” (*p* = 0.002). This baseline age difference was no longer observed following the intervention. At post-intervention, junior high school students were more likely than high school students to agree that “I look forward to growing older” (*p* = 0.023) and “Aging restricts involvement in society” (*p* = 0.005). Across other domains, including health consciousness, relevance of aging topics, and intergenerational engagement, both age groups showed improvement, but no additional significant differences were detected.

## 4. Discussion

Overall, our findings indicate that participation in the Active Aging for L.I.F.E. four-part program positively influenced adolescents’ perceptions of aging and health. Students demonstrated increased understanding of aging as a meaningful and relevant life process, greater appreciation for intergenerational engagement, and heightened awareness of health-promoting behaviors across the lifespan. At the same time, participants developed a more nuanced understanding of aging that acknowledged both opportunities and challenges, suggesting increased aging awareness rather than simplistic or stigmatized views. These findings are also consistent with lifespan developmental perspectives, which emphasize that attitudes toward aging and health are formed early and evolve across the life course [9,10]. By situating aging as a continuous and personally relevant process, the program aligns with life-course models that link early beliefs and behaviors to later-life health trajectories. Collectively, these shifts indicate that a brief, school-based intergenerational intervention can meaningfully shape adolescents’ understanding of aging and health, positioning aging as relevant to their lives now and in the future.

These findings can be understood through the lens of intergenerational contact theory, which posits that meaningful, cooperative interactions between age groups can reduce stereotypes and promote more positive attitudes toward aging [11,12]. Consistent with this framework, the program’s structure (i.e., bringing together adolescents, college students, and older adults in collaborative discussions and shared learning) likely facilitated perspective-taking, reduced social distance, and challenged age-based stereotypes. The observed shift toward more nuanced perceptions of aging suggests that participants moved beyond simplistic or deficit-based views, aligning with evidence that structured intergenerational contact can mitigate ageism and foster mutual understanding [13].

While the program demonstrated overall positive effects across the sample, additional analyses revealed meaningful variation in program-related changes by sex/gender and age group, highlighting differences in how adolescents engaged with and responded to the intervention. We found that female students demonstrated a greater increase than males in recognizing the importance of exercise across the lifespan, effectively closing an initial baseline gap in this health-related belief. Previous studies also show that female adolescents were less motivated to be physically active and had more perceived barriers to physical activity than male adolescents [14,15,16]. Our findings suggest that the program may be especially effective in increasing health behavior awareness among girls, who initially placed less importance on being physically active at any age. Female students also showed heightened post-program awareness of the relationship between personal behaviors and health, a pattern consistent with prior research demonstrating greater responsiveness to health education and health-related messaging among adolescent girls [17]. These differences may reflect gendered socialization processes, whereby girls are more often encouraged to engage in health-related behaviors, self-reflection, and interpersonal communication, while boys may be less likely to engage with topics related to aging and emotional expression [18,19]. Such patterns can shape how adolescents interpret and respond to health messaging, influencing both baseline attitudes and responsiveness to intervention content. From this perspective, the program may have been particularly effective in addressing gendered gaps by creating a space that normalizes reflection, communication, and engagement with aging-related topics across both groups.

The elimination of several baseline sex/gender differences following the intervention suggests that the Active Aging for L.I.F.E. program may have helped reduce gender-based disparities in health-related perceptions. Prior to the program, female students were less likely to report enjoying time with older adults or to endorse a sense of life purpose, while male students were less likely to perceive aging-related topics as relevant. These differences were no longer evident post-intervention. Although the program did not explicitly target constructs such as life purpose or gender norms, this convergence may reflect increased reflection, relevance, and connection fostered through the program’s intergenerational and lifespan-focused approach. Additionally, female students showed greater post-program awareness of the relationship between personal behaviors and health, while male students demonstrated increased recognition of the relevance of aging topics. Collectively, these findings suggest that the program supported more equitable health awareness among adolescents while addressing distinct baseline gaps among girls and boys.

Program effects also varied by age group, with junior high school students demonstrating stronger shifts in future-oriented attitudes toward aging than their high school counterparts. In particular, younger adolescents showed a greater increase in looking forward to growing older, consistent with developmental frameworks suggesting that early adolescence is a period marked by emerging future orientation, expanding abstract thinking, and active construction of future self-concepts [20,21,22]. This developmental period may represent a critical window for shaping beliefs about aging, as attitudes and expectations about the future are still forming [23]. Although both age groups exhibited improvements in health consciousness and aging awareness, the more pronounced attitudinal shifts among younger students, particularly in expectations for their own aging, highlight the potential value of introducing aging education earlier in the life course. These findings suggest that aging-focused health literacy interventions may be especially impactful when delivered prior to the consolidation of more stable age-related beliefs.

Importantly, these findings also contribute to the growing literature on ageism in adolescence, a developmental period during which age-related stereotypes can emerge and become internalized. Developmental and social-cognitive theories suggest that youth begin forming social category beliefs early, often incorporating societal stereotypes about age through observation and social learning [24]. Adolescents may acquire implicit or explicit biases about older adults through media portrayals, cultural norms, and limited opportunities for meaningful intergenerational contact [25,26]. Without an intervention, these perceptions can persist into adulthood and shape both interpersonal attitudes toward older adults and individuals’ own expectations of aging, consistent with stereotype embodiment theory [27]. The present findings suggest that early, structured exposure to diverse aging experiences may interrupt these processes by promoting empathy, reducing stereotypes, and encouraging adolescents to envision their own aging in more positive and realistic ways, consistent with emerging evidence on interventions to reduce ageism across the lifespan [28].

The integration of the Active Aging for L.I.F.E. framework and small group discussions on the topics between three generations (older adult, college-age, and adolescents) led to mutual respect and understanding on a broad range of topics such as the importance of social interaction, daily movement and exercise, balanced diet with high fruit and vegetable intake and low fat, sugar and sodium intake, as well as the importance of mindfulness in times of stress for personal growth and resilience. These interactions facilitated perspective-taking and narrative exchange, allowing adolescents to engage with lived experiences of aging in ways that challenge dominant social representations of older adulthood [12,13]. Through dialogue and shared storytelling, participants may have reconstructed their understanding of aging, shifting from abstract or stereotypical views to more personalized and realistic interpretations [29]. As one of the older adult L.I.F.E. leaders stated, “We are all in the same boat, we are just at different ends of it”; they resonated with the intergenerational program participants and spoke to the importance of the breakdown of an us/them agist culture. Interacting with older adults to discuss lived experiences may be uncommon for many adolescents, but these interactions can foster a deeper understanding of aging, both in others and in themselves, over time [30]. The final program “See Me at 70” reflection was a catalyst to synthesize what the adolescents had taken from the four sessions and to apply the L.I.F.E. framework to their own values and beliefs so that they could begin to see the power that they have in controlling their health and wellness outcomes across their lifespan.

Student participants were provided with a perspective that the aging process is across the lifespan, not just when an individual turns 65. This brings an understanding that the perspectives and behaviors that they adopt as an adolescent can have a positive impact throughout their lifespan. Having these conversations early allows the participants to start to think about how these topics relate to themselves, as well as to their communities. The study outcomes suggest that the participants are beginning to understand how aging relates to them right now, not just as something to think about in the future.

Through the application of the L.I.F.E. framework, the scalable program can be replicated in a variety of venues and with generational groups across the lifespan. The mix of presentations, conversations and experiential learning projects during each session can be tailored to the needs of a specific group or community. The rural context may also play a distinct role, as smaller communities often provide fewer structured opportunities for intergenerational engagement, potentially amplifying the impact of programs that intentionally facilitate these interactions [31]. In future programming, the researchers hope to take the Active Aging for L.I.F.E. program into urban schools to identify the needs and differences between adolescents growing up in rural vs. urban communities.

An intersectional perspective further enriches the interpretation of these findings. Adolescents’ perceptions of aging and health are not shaped by a single social category, such as age or gender but by the interaction of multiple social identities and structural contexts, including race/ethnicity and rurality [32]. In the present study, the largely Caucasian and rural sample may reflect shared cultural norms regarding aging, health behaviors, and intergenerational relationships, which can shape both baseline attitudes and responsiveness to intervention content [31,33]. At the same time, the observed gender differences highlight how socially constructed expectations around health, physical activity, and emotional engagement may intersect with developmental stage and cultural context to influence adolescents’ engagement with aging-related content [18,19]. From an intersectional lens, these findings suggest that program effects are not uniform but may vary across overlapping systems of identity and context. Future research should examine how these intersecting identities, particularly among more racially and ethnically diverse or urban populations, shape both baseline perceptions and intervention effectiveness. Such work is critical for ensuring that aging education initiatives are culturally responsive, contextually grounded, and equitable across diverse youth populations. For example, gender differences observed in the present study may not operate independently, but may be shaped by the intersection of gender with rural cultural norms, racial/ethnic background, and access to health resources, suggesting that adolescents’ responses to the intervention are embedded within broader social and structural contexts [32,33].

### Limitations

The study used a small, non-random sample from three rural schools and a pre–post design without a comparison group, limiting causal inference and generalizability. Although the sample included some racial and ethnic diversity, the relatively small and rural sample limited our ability to fully examine intersectional differences across groups, which should be explored in future research. Outcomes were based on self-reported data and may be subject to response bias. In addition, the study assessed changes in attitudes and perceptions, rather than actual behavior change; therefore, it is unclear whether improved aging awareness and health consciousness translated into sustained behavioral outcomes. Finally, outcomes were measured immediately post-intervention, and the longevity of observed changes is unknown. Future research should incorporate longitudinal follow-up assessments, behavioral outcome measures, and comparison groups to more fully evaluate the program’s impact on adolescent health trajectories. Nonetheless, changes in perceptions and attitudes represent an important first step in health behavior change, particularly during adolescence, when beliefs and behavioral intentions are still forming.

## 5. Conclusions

The findings of this study indicate that the Active Aging for L.I.F.E. program is an effective intergenerational intervention for improving adolescents’ perceptions of aging and health in rural communities. Participation was associated with increased aging awareness, greater recognition of health-promoting behaviors across the lifespan, and reduced sex/gender-based disparities in health-related perceptions. Stronger effects observed among younger adolescents highlight early adolescence as a critical period for fostering future-oriented thinking and aging-related health literacy. From a public health perspective, these results suggest that integrating aging education and intergenerational engagement into adolescent health programming may support earlier prevention efforts, promote lifespan health equity, and strengthen community social connectedness. Scalable interventions such as Active Aging for L.I.F.E. may represent a promising strategy for advancing population health outcomes in underserved settings.

## Figures and Tables

**Table 1:** High school and junior high school students’ demographics (*n* = 86).

Socio-Demographics	*n* (%)
Sex/Gender	
Men	33 (38)
Women	53 (62)
Race/Ethnicity	
White	38 (44)
White and African American	3 (3)
White and American Indian	5 (6)
White and Hispanic	5 (6)
White and Other	1 (1)
African American	4 (5)
American Indian	9 (11)
Hispanic	16 (19)
Asian	2 (2)
Other	2 (2)
Missing	1 (1)
Grade Level	
7th Grade	22 (26)
8th Grade	23 (27)
Freshman high school	19 (22)
Sophomore high school	9 (10)
Junior high school	9 (10)
Senior high school	4 (5)

**Table 2:** Changes in study outcomes from pre- to post-intervention (paired *t*-tests).

Statements	Pre Mean (SD)	Post Mean (SD)	t	*p*-Value
It is a privilege to grow old.	3.18 (1.33)	3.68 (1.29)	3.24	0.002
Topics related to active aging are relevant to me.	2.98 (1.04)	3.39 (1.08)	2.49	0.015
I like spending time with older adults aged 65 and older.	3.05 (1.27)	3.37 (1.27)	2.16	0.034
Connecting with others face-to-face is important to me.	3.61 (1.23)	4.01 (1.19)	2.58	0.012
Brain exercise is as important as physical exercise for my well-being.	4.18 (0.84)	4.52 (0.84)	3.00	0.004
My independence is supported by where I live.	3.41 (1.15)	3.73 (1.01)	2.11	0.039
Discussing my feelings is difficult for me.	4.15 (1.11)	3.82 (1.19)	−2.21	0.030
Aging restricts involvement in society.	2.72 (1.09)	3.12 (1.19)	2.58	0.012
I expect that as I get older, I will spend less time with friends and family.	2.43 (1.39)	2.95 (1.40)	2.79	0.007

**Table 3:** Sex/gender differences in study outcomes at pre, post, and change.

Statements	Time Point	Male (SD)	Female (SD)	*p*-Value
It is important to exercise at any age.	Pre	4.61 (0.62)	4.18 (0.97)	0.028 *
	Post	4.40 (1.04)	4.49 (0.77)	0.661
	Change	−0.18 (1.22)	0.34 (0.98)	0.047 *
I like spending time with older adults age 65 and older.	Pre	3.53 (1.17)	2.76 (1.27)	0.008 **
	Post	3.70 (1.24)	3.16 (1.31)	0.075
	Change	0.18 (1.39)	0.40 (1.23)	0.466
My life has purpose.	Pre	4.53 (0.78)	4.06 (1.03)	0.032 *
	Post	4.43 (0.90)	4.13 (1.25)	0.244
	Change	−0.11 (1.15)	0.07 (1.14)	0.528
Topics related to active aging are relevant to me.	Pre	2.53 (1.33)	3.16 (0.77)	0.009 **
	Post	3.23 (0.97)	3.55 (1.12)	0.202
	Change	0.56 (1.50)	0.31 (1.29)	0.468
My personal behaviors such as sleep and eating patterns impact my health.	Pre	4.29 (1.10)	4.48 (0.78)	0.360
	Post	4.33 (0.92)	4.69 (0.58)	0.036 *
	Change	−0.11 (1.10)	0.05 (0.95)	0.266

Note. * *p* < 0.05, ** *p* < 0.01.

## Data Availability

De-identified data may be made available upon reasonable request.
